# Flow-Guided Mimicry Covert Communication over Learned Legitimate OFDM Signal Manifolds

**DOI:** 10.3390/s26113294

**Published:** 2026-05-22

**Authors:** Qi Feng, Junyi Zhang, Mingdi Li, Li Chen

**Affiliations:** 1 The 54th Research Institute of China Electronics Technology Group Corporation (CETC), Shijiazhuang 050081, China; fq_learning@outlook.com (Q.F.);; 2 Hebei Key Laboratory of Electromagnetic Spectrum Cognition and Control, Shijiazhuang 050081, China

**Keywords:** covert communication, OFDM, normalizing flow, mimicry covert communication, legitimacy detection, latent sign modulation, robust coordinate selection, polar coding

## Abstract

Classical covert wireless communication is commonly formulated under a noise-only null hypothesis, in which a warden detects the presence of a transmission. In shared-spectrum settings with persistent legitimate traffic, however, a warden may already observe legitimate traffic and may therefore test whether an observation is statistically consistent with a legitimate signal class. Motivated by this regime, this paper studies mimicry covert communication in the post-demodulation OFDM resource-grid domain. A normalizing flow is trained on legitimate IEEE 802.11a NonHT-Data resource-grid observations, and covert bits are embedded by shared-key latent sign modulation, whose inner coordinatewise sign-flip rule preserves the standard Gaussian prior and thus the learned legitimate distribution under the ideal flow model. To improve message recovery under observation-domain perturbations, the framework further combines this inner embedding with a two-stage, two-state robustness-aware coordinate selector and a CRC-Polar outer code with reliability-weighted soft decoding. Experiments show that the coded design substantially improves message recovery over an uncoded repeated-sign baseline while keeping Willie-side discriminability low under both classifier-based and flow-density typicality tests. The study focuses on the learned post-demodulation resource-grid observation domain and leaves full over-the-air RF-chain validation for future work.

## 1. Introduction

Covert communication seeks to conceal not only message content but also the existence of transmission. Under the classical additive white Gaussian noise (AWGN) formulation, a warden distinguishes signal-plus-noise from noise alone, which leads to the square-root law: under strict detectability constraints, only $O(\sqrt{n})$ covert bits can be transmitted reliably over *n* channel uses [1,2]. This formulation has motivated extensive work on coding, power control, artificial noise, relaying, reconfigurable intelligent surfaces, and waveform shaping. Recent studies continue to refine these mechanisms in relay-assisted, RIS-assisted, and waveform-engineered settings, which confirms that the noise-only null hypothesis remains useful for many wireless security problems [3,4,5].

A different operating regime arises when the warden already observes legitimate traffic. In dense and shared-spectrum environments, the warden may not attempt full payload recovery. Instead, the task may be to determine whether an observed sample is statistically consistent with a benign traffic class in a chosen observation domain. Under this viewpoint, the covert design objective shifts from hiding energy to preserving legitimacy. The transmitter should therefore not only remain difficult to detect, but should also remain close to the distribution of legitimate signals.

This perspective differs from coarse PHY-format imitation. If the warden only checks whether a transmission resembles a generic OFDM signal, then a conventional OFDM waveform may already pass such a coarse structural check. The setting considered here imposes a more specific legitimacy requirement. The warden is assumed to judge whether an observed sample is statistically consistent with a specific class of legitimate OFDM observations rather than merely whether it has an OFDM-like structure. The goal is therefore not simply to emulate the OFDM format, but to embed a covert message while remaining close to a learned legitimate-signal manifold.

This legitimacy-based regime gives rise to mimicry covert communication, in which the legitimate signal class serves as the cover source. In distributional terms, the covert encoder should induce stego observations that remain compatible with the cover distribution while carrying recoverable hidden information. This view is consistent with information-theoretic steganography, where security is characterized by the warden’s ability to distinguish cover and stego distributions [6]. In the OFDM resource-grid domain considered here, the cover source is the class of protocol-conforming post-demodulation observations, and mimicry means statistical legitimacy with respect to the learned distribution of that class.

Prior work has explored biomimetic and cover-source-inspired signaling, where covert transmissions are designed to resemble benign acoustic, biological, radio, or hardware-noise patterns [7,8]. Examples include acoustic schemes that imitate biological sounds, GAN-based dolphin-whistle generation for underwater communication [9], wireless steganographic schemes based on hardware-noise-like or cover-waveform-like structures [10], and multi-carrier phase randomization for noise-like covert signaling [11]. Recent surveys further describe the broader development of learning-assisted and intelligent covert communication [12]. These studies shift the design objective from energy concealment toward distributional resemblance. However, their cover sources are typically specified by a particular acoustic template, hardware-noise mechanism, or handcrafted waveform construction. They do not directly address the case in which the cover source is a high-dimensional distribution of OFDM resource-grid observations learned from data.

Generative modeling provides a natural way to represent such a cover distribution. Among deep generative models, normalizing flows are especially suitable because they provide exact density evaluation, exact latent inference, and exact synthesis through invertible mappings [13,14,15]. These properties are useful for distribution-oriented covert communication: the learned likelihood characterizes legitimate observations, the inverse map synthesizes samples from latent variables, and the forward map supports receiver-side latent recovery. When the latent prior is symmetric, an embedding rule can be constructed to preserve the prior exactly, thereby preserving the learned legitimate distribution under the ideal flow model. Recent progress in wireless generative modeling further supports the use of data-driven signal manifolds for radio synthesis, waveform generation, and channel estimation [16,17]. Normalizing flows have also been used for invertible information hiding outside the OFDM setting [18]. In contrast to phase-randomized or noise-like waveform construction, the present work considers a learned distribution of legitimate OFDM resource-grid observations. The embedding rule must therefore preserve statistical legitimacy, maintain shared-key synchronization, and remain recoverable after observation-domain perturbations. In parallel, RF anomaly-detection studies indicate that legitimacy can be treated as a statistical detection problem over observed signal distributions rather than only as a semantic or protocol-level decision [19].

Building on this perspective, this paper studies learned-manifold mimicry covert communication in a post-demodulation OFDM resource-grid observation domain. Legitimate samples are obtained from protocol waveform generation followed by OFDM demodulation of the data field, and a normalizing flow is trained to model the resulting resource-grid distribution. This formulation casts covert embedding as distribution-preserving synthesis on a learned legitimate manifold and supports the proposed flow-guided embedding framework.

On top of this learned model, covert information is embedded through a shared-key latent sign-modulation rule. A secret key and frame index jointly determine a reference latent template, and selected latent coordinates are either preserved or sign-flipped to encode covert bits. Because coordinatewise sign flips preserve a zero-mean isotropic Gaussian prior exactly, this inner embedding preserves the learned legitimate distribution under the ideal latent model. In practice, however, legitimacy preservation alone does not guarantee reliable message recovery. Observation-domain perturbations are reshaped by the nonlinear flow into coordinate-dependent and state-dependent latent distortions, which can destabilize the recovered signs and create a gap between distribution preservation and communication reliability.

To bridge this gap, the proposed implementation combines the prior-preserving inner embedding with a two-stage, two-state robustness-aware coordinate selector and a CRC-Polar outer code with reliability-weighted soft decoding. Polar coding provides the underlying capacity-achieving code construction, while list decoding supplies the practical soft-decision recovery mechanism adopted here [20,21]. The selector identifies latent coordinates whose signs remain reliable under both covert-bit states after inverse-flow synthesis, perturbation, and forward-flow recovery, while the outer code converts these imperfect sign channels into a stronger message-level link without altering the inner distribution-preserving rule.

The contributions of this paper are threefold. First, the paper formulates covert communication as a legitimacy-based learned-manifold mimicry problem, in which the warden tests statistical consistency with a learned distribution of benign OFDM observations rather than silence versus transmission. Second, it introduces a flow-guided latent sign-modulation rule that embeds covert bits while preserving the Gaussian latent prior exactly under the ideal model. Third, it identifies a geometry-induced reliability bottleneck caused by observation-domain perturbations and develops a coded architecture that combines two-state robustness-aware coordinate selection with CRC-Polar outer coding and reliability-weighted soft decoding. The analysis further separates the distributional legitimacy guaranteed by the inner sign rule from the message-level reliability obtained through geometry-aware selection and outer coding.

The remainder of this paper is organized as follows. Section 2 presents the system model and problem formulation. Section 3 describes the proposed scheme. Section 4 develops the theoretical analysis. Section 5 reports the experimental results. Section 6 concludes the paper.

## 2. System Model and Problem Formulation

**Figure 1:**
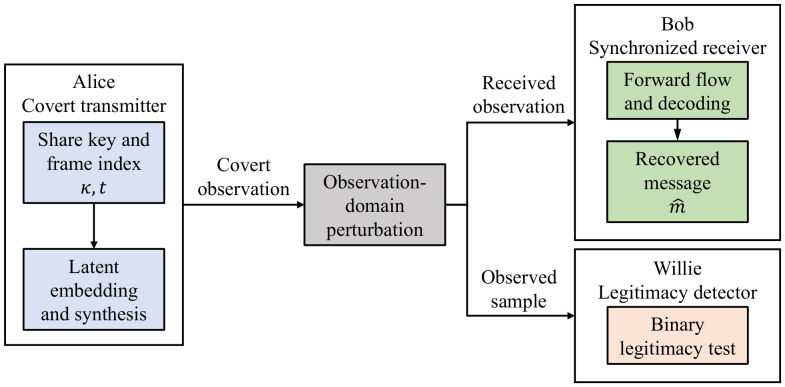
System-level interaction model for learned-manifold mimicry covert communication in the adopted frequency-domain OFDM resource-grid observation domain. Alice uses the shared key and frame index to perform latent embedding and inverse-flow synthesis of covert observations. After perturbation in the observation domain, Bob applies forward-flow recovery and decoding to reconstruct the hidden message, while Willie performs a binary legitimacy test on the same observation representation.

This section formalizes the learned-manifold mimicry covert communication problem studied in this paper. It first defines the legitimate observation space and the corresponding learned manifold in the adopted frequency-domain OFDM resource-grid representation, and then specifies the three-party interaction, the legitimacy-detection viewpoint, the observation model, and the resulting design objectives. Figure 1 summarizes the system-level interaction in the adopted observation domain, including shared-key latent embedding and synthesis at Alice, observation-domain perturbation, receiver-side forward-flow recovery and decoding at Bob, and binary legitimacy testing at Willie.

### 2.1. Legitimate Signal Space and Learned Manifold

Let $x\in R^{D}$ denote a real-valued representation of a legitimate OFDM observation in the adopted frequency-domain resource-grid domain, where *D* is the ambient dimension of that representation. Here, x denotes a generic sample drawn from the class of benign protocol-conforming OFDM data-field observations, and the implementation-specific construction of this representation is described in Section 5. Let $p_{\mathrm{legit}}\left(x\right)$ denote the unknown distribution of legitimate observations in this space.

In the adopted representation, legitimacy is treated as a statistical property of the observation. A sample is legitimate when it is compatible with the target class of benign OFDM resource-grid observations captured by the learned frequency-domain manifold; payload semantics and public decodability are not part of this criterion.

A normalizing flow learns an invertible mapping z=F(x) and $x=G\left(z\right)=F^{-1}\left(z\right)$, where the latent variable $z\in R^{D}$ follows a simple prior distribution $p_{0}\left(z\right)$, chosen here as a standard Gaussian N(0,I). The learned density is given by

(1) $$\log p_{\theta }\left(x\right)=\log p_{0}\left(F\left(x\right)\right)+\log\left|\det J_{F}\left(x\right)\right|,$$

where $J_{F}\left(x\right)$ denotes the Jacobian of *F*. After training, *G* synthesizes observations that follow the learned legitimate distribution, whereas *F* maps observed samples back into latent space. In this sense, the learned manifold viewpoint adopted in this paper is operational rather than purely geometric: legitimate observations are those that remain statistically compatible with the distribution captured by the trained flow.

### 2.2. Participants and Detection Viewpoint

Alice seeks to transmit a covert message to Bob while ensuring that the constructed covert observation remains statistically legitimate to Willie in the adopted observation domain. The key distinction from classical covert communication lies in the null hypothesis available to Willie. Instead of testing

(2) $$H_{0}:\mathrm{noise}\;\mathrm{only},\;H_{1}:\mathrm{signal}\;\mathrm{plus}\;\mathrm{noise},$$

we consider a legitimacy-based viewpoint in which Willie effectively tests

(3) $$H_{0}:y∼q_{\mathrm{legit}},\;H_{1}:y∼q_{\mathrm{covert}},$$

where $q_{\mathrm{legit}}$ and $q_{\mathrm{covert}}$ denote the observed-sample distributions in the adopted observation domain under the perturbation model, corresponding respectively to legitimate and covert generation.

Under this viewpoint, the design objective is not merely to reduce observable energy, but to keep $q_{\mathrm{covert}}$ close to $q_{\mathrm{legit}}$ in a distributional sense while still conveying hidden information. This criterion is stronger than coarse waveform-format recognition: the relevant question is not whether an observation can be recognized as OFDM in a superficial structural sense, but whether it remains statistically consistent with the target class of legitimate OFDM observations while carrying a recoverable covert message. The problem studied in this paper is therefore a mimicry covert communication problem, in which covert signaling must remain compatible with a learned manifold of benign observations rather than merely evade a silence-versus-transmission detector.

### 2.3. Transmission and Observation Model

In the adopted observation-domain formulation, Alice first generates a clean covert observation x from the learned legitimate manifold, and both Bob and Willie observe the perturbed sample

(4) $$y=x+n,\;n∼N(0,\sigma^{2}I).$$

This additive model serves as the basic perturbation channel through which robustness and detectability are studied. Accordingly, Willie’s legitimacy test is posed on the observed sample y rather than on the clean pre-perturbation sample x.

Two perturbation models are considered. In the primary model, noise is added directly to the raw, unstandardized OFDM resource-grid observation vector. This setting, referred to throughout the paper as de-normalized sample-space perturbation, operates in the native scale of the adopted observation representation and serves as the main evaluation setting for the proposed observation-domain formulation. It preserves the geometry induced by inverse-flow synthesis and forward-flow recovery and therefore captures the practical sign-instability effects that arise when perturbations are applied in sample space.

In the secondary model, used mainly for algorithmic diagnosis, noise is injected after the observation vector has been standardized to zero mean and unit variance and before it is mapped back through *F*. This setting, referred to as normalized-space perturbation, suppresses the scale distortion induced by the native observation coordinates and thereby provides a controlled reference for isolating the geometry-dependent reliability loss analyzed in Section 4. The comparison between these two models helps distinguish effects caused by the learned flow geometry from those tied to the native scaling of the observation representation.

### 2.4. Design Objectives

Three interrelated objectives govern the covert-signaling problem studied in this paper. The first is legitimacy preservation: generated observations should remain statistically distributed as legitimate OFDM observations in the adopted domain. The second is receiver reliability: Bob should recover the hidden message with low error probability and high effective throughput. The third is shared-key synchronization: covert information should be recoverable only by a receiver possessing the shared secret key and the agreed frame-index schedule.

The central tension studied in this paper lies between the first two objectives. Prior-preserving latent modulation addresses legitimacy under the ideal model by maintaining compatibility with the Gaussian latent prior and, consequently, with the learned legitimate distribution. Reliable communication, however, depends on whether the selected latent coordinates remain stable binary sign channels after inverse-flow synthesis, sample-space perturbation, and forward-flow recovery. The resulting problem therefore involves two related but distinct requirements: preserving statistical legitimacy with respect to the learned observation manifold and maintaining reliable message recovery under observation-domain perturbations.

## 3. Proposed Flow-Guided Mimicry Covert Communication Scheme

**Figure 2:**
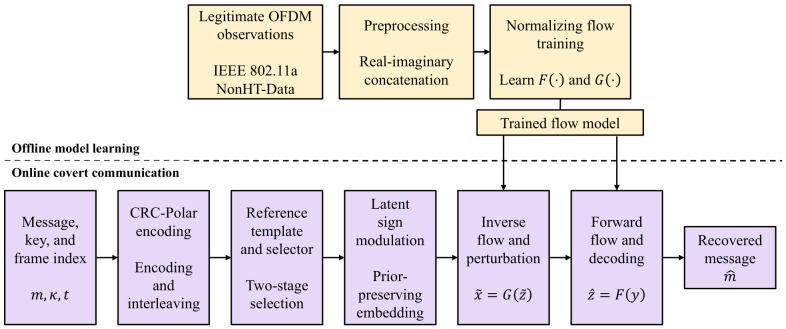
End-to-end framework of the proposed flow-guided mimicry covert communication scheme. Legitimate OFDM observations are preprocessed and used to train a normalizing flow offline. During online covert communication, the hidden message is outer-coded, mapped onto selected latent coordinates through shared-key sign modulation, synthesized by the inverse flow, perturbed in the observation domain, and recovered at the receiver through forward-flow processing and decoding.

This section presents the proposed flow-guided mimicry covert communication scheme. As summarized in Figure 2, the overall design consists of two coupled parts: offline learning of a normalizing flow from legitimate OFDM observations and online covert communication over the learned observation manifold. Within the online phase, the proposed implementation combines four components: shared-key latent template generation, two-state robustness-aware coordinate selection, CRC-Polar outer coding with interleaved sign modulation, and receiver-side soft decoding. Together, these components are designed to preserve statistical legitimacy at the inner embedding level while improving message recovery under observation-domain perturbations.

### 3.1. Shared-Key Latent Template Generation

For each frame index *t*, Alice and Bob use a shared secret key κ together with the frame index *t* to deterministically generate a reference latent template

$$z^{\mathrm{ref}}\left(\kappa ,t\right)∼N\left(0,I\right),$$

which is implemented in practice by a pseudorandom generator seeded by (κ,t). Because the generation rule is deterministic once the seed is fixed, the same reference template can be reconstructed at the receiver, whereas Willie cannot reproduce it without knowledge of the shared key.

The template $z^{\mathrm{ref}}\left(\kappa ,t\right)$ serves as the legitimate latent reference before covert embedding. Covert information is introduced only by modifying a selected subset of its coordinates, while all remaining coordinates are preserved. This shared-key latent template therefore provides a synchronized reference for both embedding and recovery and, at the same time, anchors the covert construction to the learned Gaussian latent prior.

### 3.2. Two-State Robustness-Aware Coordinate Selection

A central design problem is to determine which latent coordinates should carry covert bits. Here, a coordinate refers to one scalar component of the latent vector. For $z\in R^{D}$, coordinate *i* denotes the *i*th entry, written as ${\left[z\right]}_{i}$.

A natural initial idea is to prefer coordinates with large absolute values in the reference latent template, namely large $\left|{\left[z^{\mathrm{ref}}\left(\kappa ,t\right)\right]}_{i}\right|$. The intuition is that a coordinate far from zero should be less likely to experience a sign reversal under perturbation. Although useful, this criterion is incomplete in the present system. Recovery does not occur directly from the clean reference latent vector. Instead, it takes place only after the chain

$$z^{\mathrm{ref}}\left(\kappa ,t\right)\to G\left(\cdot \right)\to \text{observation-domain}\;\mathrm{perturbation}\to F\left(\cdot \right).$$

Because *G* and *F* are nonlinear, perturbations that are simple in observation space generally become coordinate-dependent after they are mapped back into latent space. Consequently, two coordinates with similar values of $\left|{\left[z^{\mathrm{ref}}\left(\kappa ,t\right)\right]}_{i}\right|$ can exhibit markedly different sign reliabilities after the round trip, so a magnitude-only rule is insufficient.

A second issue arises from the binary use of each selected coordinate. If the transmitted coded bit is 0, the corresponding reference sign is preserved; if the transmitted coded bit is 1, the corresponding reference sign is flipped. A coordinate is therefore useful only if it remains reliable in both states. A selector that evaluates only one state may retain coordinates that are stable when the transmitted bit is 0 but fragile when the transmitted bit is 1. For this reason, the proposed selector explicitly evaluates both states.

To balance reliability and computational complexity, the implementation adopts a two-stage procedure. The first stage performs coarse screening, and the second stage performs binary-state verification.

In the first stage, an eligible set is formed by thresholding $\left|{\left[z^{\mathrm{ref}}\left(\kappa ,t\right)\right]}_{i}\right|$, which removes coordinates whose reference signs are intrinsically too close to zero. From this eligible set, the selector keeps a shortlist of candidate coordinates and probes their stability under the reference state. For each candidate coordinate, the transmitter synthesizes the corresponding observation through *G*, injects perturbation in the chosen evaluation space, maps the perturbed observation back through *F*, and records whether the recovered sign agrees with the original reference sign. Repeating this procedure over several trials yields a coarse estimate of sign stability together with a margin-type statistic that reflects how confidently the recovered value remains on the correct side of zero. These quantities are combined into a first-stage score and used only to discard weak coordinates before the more expensive second-stage test.

Specifically, let ${\hat{z}}^{\left(\ell ,+\right)}$ denote the recovered latent vector in the *ℓ*th first-stage positive-state probe. With $L_{1}$ first-stage probe trials, the implemented shortlist score is

(5) $$\begin{array}{cc} {\bar{p}}_{i}^{\left(+\right)} & =\frac{1}{L_{1}}\sum_{\ell =1}^{L_{1}}1\;\left\{\mathrm{sign}\;\left({\left[{\hat{z}}^{\left(\ell ,+\right)}\right]}_{i}\right)=\mathrm{sign}\;\left({\left[z^{\mathrm{ref}}\right]}_{i}\right)\right\}, \end{array}$$

(6) $$\begin{array}{cc} {\bar{m}}_{i}^{\left(+\right)} & =\frac{1}{L_{1}}\sum_{\ell =1}^{L_{1}}\frac{\mathrm{sign}\;\left({\left[z^{\mathrm{ref}}\right]}_{i}\right){\left[{\hat{z}}^{\left(\ell ,+\right)}\right]}_{i}}{|{\left[z^{\mathrm{ref}}\right]}_{i}|+\epsilon_{\mathrm{num}}}, \end{array}$$

(7) $$\begin{array}{cc} q_{i} & =0.75\;{\bar{p}}_{i}^{\left(+\right)}+0.15\;\tanh\left({\bar{m}}_{i}^{\left(+\right)}\right)+0.10\;\tanh\;\left(\frac{|{\left[z^{\mathrm{ref}}\right]}_{i}|}{3}\right). \end{array}$$

Here ${\bar{p}}_{i}^{\left(+\right)}$ is the empirical first-stage positive-state sign-stability estimate, ${\bar{m}}_{i}^{\left(+\right)}$ is the corresponding normalized signed margin, and the last term weakly favors coordinates whose reference latent magnitude is farther from the zero-sign boundary.

In the second stage, the best first-stage candidates are re-evaluated under both covert states. For a candidate coordinate *i*, let the positive state denote the case in which the reference sign is preserved, and let the negative state denote the case in which the reference sign is flipped. The selector then estimates the empirical sign-preservation probabilities

(8) $$\begin{array}{cc} p_{i}^{\left(+\right)} & =\mathrm{Pr}\;\left(\mathrm{sign}\left({\left[\hat{z}\right]}_{i}\right)=\mathrm{sign}\left({\left[z^{\mathrm{ref}}\left(\kappa ,t\right)\right]}_{i}\right)|\mathrm{positive}\;\mathrm{state}\right), \end{array}$$

(9) $$\begin{array}{cc} p_{i}^{\left(-\right)} & =\mathrm{Pr}\;\left(\mathrm{sign}\left({\left[\hat{z}\right]}_{i}\right)=-\mathrm{sign}\left({\left[z^{\mathrm{ref}}\left(\kappa ,t\right)\right]}_{i}\right)|\mathrm{negative}\;\mathrm{state}\right), \end{array}$$

where the probabilities are estimated by repeated perturb-and-recover trials. A coordinate is retained only if it is reliable in both states, and its final utility is therefore defined conservatively as

$$r_{i}=\min\left\{p_{i}^{\left(+\right)},\;p_{i}^{\left(-\right)}\right\}.$$

This minimum operator prevents the selector from favoring coordinates that are highly reliable for one bit value but weak for the other, which would otherwise create an asymmetric binary channel.

The final selected set is obtained by ranking candidates according to $r_{i}$ and retaining the strongest coordinates. These selected coordinates are precisely those whose signs remain stable after inverse-flow synthesis, perturbation in the chosen observation space, and forward-flow recovery, regardless of which covert state is used. The selector therefore plays two roles simultaneously: it identifies the latent coordinates most suitable for covert embedding, and it provides the empirical reliability values later used in soft decoding.

The selector is implemented as a deterministic function of the shared reference template $z^{\mathrm{ref}}\left(\kappa ,t\right)$, the trained flow, the public selector hyperparameters, and a prescribed probe schedule.

For coordinate *i*, probe state s∈{+,−}, and probe trial *ℓ*, the perturbation realization used in the probe is generated by a fixed pseudorandom mapping

(10) $$\eta_{i,s,\ell }=P_{\sigma_{\mathrm{sel}}}\left(\kappa ,t,i,s,\ell \right),$$

where $P_{\sigma_{\mathrm{sel}}}\left(\cdot \right)$ denotes the deterministic probe generator with public selector salt $\sigma_{\mathrm{sel}}$. Thus, for a given key κ, frame index *t*, trained flow, and selector configuration, the same candidate coordinate receives the same probe perturbations at Alice and Bob. The selector configuration uses a candidate pool of 4096 coordinates, a probe pool of 512 candidates, a minimum latent-amplitude threshold of 1.8, a probe SNR of 10 dB, and four probe trials for each evaluated coordinate.

Consequently, Bob can recompute the same selected-coordinate set and the same empirical reliabilities locally; no side information about the selected coordinates is transmitted within the covert payload.

### 3.3. CRC-Polar Outer Coding and Interleaved Sign Modulation

The uncoded reference implementation maps one message bit to a small group of repeated sign channels and then applies hard voting at the receiver. The proposed coded scheme instead treats each selected coordinate as a single coded bit-channel and places a short outer code on top of the same sign-preserving inner modulation.

Let $m\in {\left\{0,1\right\}}^{K_{\mathrm{msg}}}$ denote the covert message. Alice first appends a CRC and then applies a length-$N_{\mathrm{polar}}$ Polar encoder, which produces $c\in {\left\{0,1\right\}}^{N_{\mathrm{polar}}}$. The implementation adopts a short CRC-Polar concatenation with CRC-aided successive-cancellation list (CA-SCL) decoding [20,21]. A secret-key interleaver Π(κ,t) permutes the coded bits so that adjacent Polar positions are decoupled from adjacent selected coordinates.

Let $I=\{i_{1},\ldots ,i_{N_{\mathrm{polar}}}\}$ denote the selected coordinates, and let ${\tilde{c}}_{j}=c_{\Pi (j)}$ denote the interleaved coded bit assigned to coordinate $i_{j}$. Alice then applies coordinatewise sign modulation:

(11) $${\left[\tilde{z}\right]}_{i_{j}}=\left\{\begin{array}{cc} {\left[z^{\mathrm{ref}}\left(\kappa ,t\right)\right]}_{i_{j}}, & {\tilde{c}}_{j}=0,\\ -{\left[z^{\mathrm{ref}}\left(\kappa ,t\right)\right]}_{i_{j}}, & {\tilde{c}}_{j}=1. \end{array}\right.$$

All non-selected coordinates remain unchanged. The modulated latent vector $\tilde{z}$ is mapped into observation space through

$$\tilde{x}=G\left(\tilde{z}\right),$$

and the resulting covert observation is evaluated under the chosen perturbation model. Importantly, the outer code changes only the sign pattern assigned to the selected coordinates; it does not modify the prior-preserving inner sign-flip rule itself.

### 3.4. Receiver Processing and Soft Decoding

Bob receives the perturbed observation y and computes

$$\hat{z}=F\left(y\right).$$

Because the coordinate selector is deterministic given the shared key, frame index, trained flow, and public probe schedule, Bob can locally reproduce the selected-coordinate set I together with the corresponding reliability values $r_{j}$; these quantities do not need to be conveyed as side information.

For each selected coordinate $i_{j}$, Bob forms the local continuous statistic

(12) $$s_{j}=\frac{{\left[\hat{z}\right]}_{i_{j}}{\left[z^{\mathrm{ref}}\left(\kappa ,t\right)\right]}_{i_{j}}}{\left|{\left[z^{\mathrm{ref}}\left(\kappa ,t\right)\right]}_{i_{j}}\right|+\epsilon_{\mathrm{num}}},$$

which is positive when the observation supports the unflipped state and negative when it supports the flipped state. Here $\epsilon_{\mathrm{num}}$ is a small numerical stabilizer used only to avoid division by zero; it is distinct from the residual carrier-frequency-offset parameter $\epsilon_{\mathrm{cfo}}$ used later in Section 5.5. The two-state selector also provides an empirical coordinate reliability $r_{j}\in \left(0,1\right)$, from which Bob constructs the reliability weight

$$\alpha_{j}=\log\frac{r_{j}}{1-r_{j}}.$$

The resulting soft bit metric is

(13) $$L_{j}=\mathrm{clip}\;\left(\beta \;\alpha_{j}\;s_{j},\;-L_{\max},\;L_{\max}\right),$$

where β is a global scaling parameter and $L_{\max}$ is an implementation-level clipping threshold used for numerical stability. All coded experiments use β=1.0 and $L_{\max}=20.0$. After deinterleaving, the LLR vector is passed to a CA-SCL Polar decoder. If the CRC check fails, the frame is declared an erasure. This CRC-Polar layer is the main distinction between the coded scheme and the original repetition-sign baseline retained for reference.

The complete procedure of the proposed scheme is summarized in Algorithm 1.
**Algorithm 1** Proposed Flow-Guided Mimicry Covert Communication Procedure**Require:** Legitimate OFDM dataset, shared secret key κ, frame index *t*, covert message m**Ensure:** Recovered covert message $\hat{m}$ or declared erasure   **Phase 1: Offline Training** 1:Train a normalizing flow on legitimate OFDM resource-grid observations 2:Obtain the forward map *F* and inverse map $G=F^{-1}$   **Phase 2: Transmitter Processing** (for frame index *t*) 3:Generate the reference latent template $z^{\mathrm{ref}}\left(\kappa ,t\right)$ from κ and *t* 4:Form a candidate coordinate pool by magnitude thresholding and coarse local-stability probing 5:Evaluate shortlisted candidates under both covert states by repeated perturb-and-recover trials 6:Rank the candidates by two-state reliability 7:Select the top-$N_{\mathrm{polar}}$ coordinates 8:Encode m by CRC-Polar coding to obtain c 9:Apply key-driven interleaving Π(κ,t) to c10:Map the interleaved bits onto the selected coordinates of $z^{\mathrm{ref}}\left(\kappa ,t\right)$ by latent sign modulation to obtain $\tilde{z}$11:Synthesize the covert observation $\tilde{x}=G\left(\tilde{z}\right)$12:Generate the perturbed observation from $\tilde{x}$ under the chosen perturbation model   **Phase 3: Receiver Processing**13:Receive y and compute $\hat{z}=F\left(y\right)$14:Reconstruct $z^{\mathrm{ref}}\left(\kappa ,t\right)$15:Recompute the selected set I and empirical reliabilities $r_{j}$16:Form $\alpha_{j}=\log\;\left(r_{j}/\left(1-r_{j}\right)\right)$17:**for** each selected coordinate $i_{j}$ **do**18:    Compute the local continuous statistic $s_{j}$19:    Form the soft metric $L_{j}=\mathrm{clip}\left(\beta \alpha_{j}s_{j},-L_{\max},\;L_{\max}\right)$20:**end for**21:Deinterleave the soft metrics L using $\Pi^{-1}\left(\kappa ,t\right)$22:Apply CRC-aided successive-cancellation list decoding23:**if** the CRC check passes **then**24:    **return** recovered message $\hat{m}$25:**else**26:    **return** erasure27:**end if**

This algorithmic summary highlights how offline flow learning and online covert communication are coupled in the proposed framework.

## 4. Theoretical Analysis

This section clarifies the theoretical basis of the proposed scheme from two complementary perspectives. First, it shows that the latent sign-modulation rule, or equivalently the embedding scheme conditional on a fixed selected-coordinate set and sign pattern, preserves the standard Gaussian prior exactly and therefore preserves the learned legitimate distribution under the ideal flow model. Second, it explains why reliable message recovery is nevertheless nontrivial under observation-domain perturbations: even when legitimacy is preserved at the distributional level, the nonlinear geometry of the learned flow can distort sign stability after inverse-flow synthesis and forward-flow recovery.

### 4.1. Exact Prior Preservation Under Sign Modulation

The principal theoretical advantage of latent sign modulation is that it preserves the standard Gaussian prior exactly.

**Proposition** **1.** *Let $z^{\mathrm{ref}}∼N\left(0,I\right)$, and let D be a diagonal matrix whose diagonal entries belong to {+1,−1}. Define*

$$\tilde{z}=Dz^{\mathrm{ref}}.$$

*Then $\tilde{z}$ obeys the same distribution,*

$$\tilde{z}∼N\left(0,I\right).$$

**Proof.**  Because $\tilde{z}=Dz^{\mathrm{ref}}$ is a linear transformation of the jointly Gaussian vector $z^{\mathrm{ref}}$, the vector $\tilde{z}$ is also jointly Gaussian. Its first two moments are

(14) $$\begin{array}{cc} E[\tilde{z}] & =DE[z^{\mathrm{ref}}]=0, \end{array}$$

(15) $$\begin{array}{cc} \mathrm{Cov}(\tilde{z}) & =D\;\mathrm{Cov}\left(z^{\mathrm{ref}}\right)\;D^{T}=DID^{T}=I, \end{array}$$

where the last equality follows from $DD^{T}=I$, which holds because D is a diagonal matrix with entries in {+1,−1}. Since a Gaussian distribution is uniquely determined by its mean and covariance, it follows that $\tilde{z}∼N\left(0,I\right)$. □

**Corollary** **1.** *Let $x=G(z^{\mathrm{ref}})$ and $\tilde{x}=G\left(\tilde{z}\right)$ be generated by the same invertible flow. Under the ideal model,*

$$\tilde{x}\overset{d}{=}x.$$

**Proof.**  By Proposition 1, $\tilde{z}\overset{d}{=}z^{\mathrm{ref}}$. Since *G* is a fixed deterministic mapping, applying *G* to identically distributed inputs yields identically distributed outputs. Hence

$$G\left(\tilde{z}\right)\overset{d}{=}G\left(z^{\mathrm{ref}}\right).$$

□

Therefore, for any fixed diagonal sign matrix with entries in +1,−1, the latent sign-modulation rule preserves the learned legitimate distribution exactly when evaluated before additive perturbation.

This exact prior-preservation result establishes the ideal distributional legitimacy of the inner embedding rule. In particular, any degradation observed under observation-domain perturbations must arise not from a mismatch of the latent prior itself, but from the interaction between perturbation and the local geometry of the learned flow.

### 4.2. Geometry-Induced Reliability Loss Under Observation-Domain Perturbations

Suppose the constructed covert observation is $\tilde{x}=G\left(\tilde{z}\right)$ and the receiver observes

$$y=\tilde{x}+n.$$

The recovered latent vector is then

$$\hat{z}=F\left(y\right)=F\left(\tilde{x}+n\right).$$

A first-order expansion of *F* around $\tilde{x}$ yields

(16) $$\hat{z}\approx \tilde{z}+J_{F}\left(\tilde{x}\right)n,$$

where $J_{F}\left(\tilde{x}\right)$ denotes the Jacobian of the forward flow at the synthesized observation.

Equation (16) reveals the central reliability bottleneck. Even if n is isotropic Gaussian in observation space, the induced perturbation in latent space is filtered by the local Jacobian $J_{F}\left(\tilde{x}\right)$. As a consequence, the effective latent perturbation is generally anisotropic and coordinate-dependent after recovery through the learned flow. Sign reliability therefore varies across latent coordinates, even when the observation-space perturbation is statistically homogeneous.

A second implication is state dependence. The local Jacobian is evaluated at $\tilde{x}=G\left(\tilde{z}\right)$, and $\tilde{z}$ itself depends on the chosen sign assignment. Hence the perturbation seen by a coordinate may differ between the two covert states even when the same coordinate is used. In other words, a coordinate that is stable when the reference sign is preserved need not remain equally stable when the sign is flipped.

This local analysis clarifies the central implication of the proposed scheme. Exact prior preservation guarantees ideal distributional legitimacy before perturbation, but it does not imply robust message recovery after perturbation in the observation domain. Communication reliability is therefore governed by the local geometry of the learned flow rather than by prior preservation alone.

### 4.3. State-Dependent Sign Reliability and Two-State Coordinate Utility

For a selected coordinate *i*, define the local latent sign margin

(17) $$\gamma_{i}\left(\tilde{x}\right)=\frac{\left|{\left[\tilde{z}\right]}_{i}\right|}{\sqrt{\mathrm{Var}({\left[J_{F}\left(\tilde{x}\right)n\right]}_{i})}}.$$

Under the first-order model in (16), the numerator measures the distance of the clean latent coordinate from the decision boundary at zero, while the denominator measures the standard deviation of the induced perturbation along that coordinate. A larger value of $\gamma_{i}\left(\tilde{x}\right)$ therefore indicates a smaller local probability of sign reversal.

Because both $\tilde{x}$ and $J_{F}\left(\tilde{x}\right)$ depend on the covert state, the corresponding margin is also state-dependent. For this reason, it is useful to denote the state-specific margin by $\gamma_{i}^{\left(s\right)}$ for s∈{+,−}, where + refers to the sign-preserving state and − refers to the sign-flipped state. This suggests that a magnitude-only or single-state criterion is generally insufficient: a coordinate may appear strong under one state while remaining fragile under the other.

The following proposition makes this local interpretation explicit.

**Proposition** **2.** *Under the first-order approximation in *(16) *and Gaussian observation-space noise $n∼N(0,\sigma^{2}I)$, the conditional sign-flip probability at coordinate i in covert state s∈{+,−} is approximated by*

(18) $$P_{\mathrm{err},i}^{\left(s\right)}\approx Q\;\left(\gamma_{i}^{\left(s\right)}\right),$$

*where Q(·) is the Gaussian tail function and $\gamma_{i}^{\left(s\right)}$ is the state-dependent margin defined in* (17)*.*

**Proof.**  Under (16), the recovered coordinate satisfies

$${\left[\hat{z}\right]}_{i}\approx {\left[\tilde{z}\right]}_{i}+{\left[J_{F}\left(\tilde{x}\right)n\right]}_{i}.$$

The perturbation term is Gaussian with zero mean and variance $\mathrm{Var}({\left[J_{F}\left(\tilde{x}\right)n\right]}_{i})$. A sign error occurs when the perturbation drives ${\left[\hat{z}\right]}_{i}$ across zero. Under this local Gaussian model, the sign-flip probability is therefore given by the Gaussian tail associated with the normalized distance from ${\left[\tilde{z}\right]}_{i}$ to zero, namely $Q(\gamma_{i}^{\left(s\right)})$. □

Proposition 2 should be interpreted as a local approximation rather than as a global characterization of the nonlinear channel. Its value is to explain why sign reliability depends jointly on the latent magnitude and on the local Jacobian-induced perturbation geometry. In particular, two coordinates with similar absolute values may have substantially different sign reliabilities if their local flow geometries differ.

This state dependence motivates the use of two empirical reliabilities, $p_{i}^{\left(+\right)}$ and $p_{i}^{\left(-\right)}$, corresponding to the sign-preservation probabilities under the two covert states. For a binary sign channel built from coordinate *i*, the most fragile state determines the worst-case reliability. This motivates the conservative utility

$$U_{i}=\min\left\{p_{i}^{\left(+\right)},\;p_{i}^{\left(-\right)}\right\}.$$

**Proposition** **3.** *Let a binary sign channel based on coordinate i have state-dependent sign-preservation probabilities $p_{i}^{\left(+\right)}$ and $p_{i}^{\left(-\right)}$. Then the worst-case bit error probability of that coordinate is*

$$\varepsilon_{i}^{\max}=1-\min\left\{p_{i}^{\left(+\right)},\;p_{i}^{\left(-\right)}\right\}=1-U_{i}.$$

*Consequently, ranking coordinates by $U_{i}$ directly ranks them by worst-case bit reliability.*

**Proof.**  When bit 0 is transmitted, the coordinate error probability is $1-p_{i}^{\left(+\right)}$; when bit 1 is transmitted, the coordinate error probability is $1-p_{i}^{\left(-\right)}$. The worst-case bit error probability is therefore

$$\varepsilon_{i}^{\max}=\max\left\{1-p_{i}^{\left(+\right)},\;1-p_{i}^{\left(-\right)}\right\}=1-\min\left\{p_{i}^{\left(+\right)},\;p_{i}^{\left(-\right)}\right\}.$$

Since $U_{i}=\min\left\{p_{i}^{\left(+\right)},\;p_{i}^{\left(-\right)}\right\}$, it follows that $\varepsilon_{i}^{\max}=1-U_{i}$. □

Proposition 3 provides the formal justification for the two-state selector used in Section 3.2. A selector based only on one state can rank a coordinate highly even if its opposite-state reliability is poor, whereas the utility $U_{i}$ controls the worst-case binary reliability by construction. After ranking and truncation, the retained utility of the *j*th selected coordinate is denoted by $r_{j}$.

The receiver uses $r_{j}$ not only for coordinate selection but also as a soft confidence factor. Accordingly, the heuristic LLR in (13) combines two pieces of information: the instantaneous sign evidence from the recovered latent coordinate and the long-term state-aware reliability estimated by the two-state probe. This construction is intentionally heuristic rather than a fully matched log-likelihood for the induced nonlinear channel, but it is consistent with the role of the selected coordinates as unequal-quality binary sign channels.

### 4.4. Legitimacy Preservation Versus Communication Reliability

The preceding analysis shows that legitimacy preservation and communication reliability are related but fundamentally distinct notions in the present problem.

Legitimacy is governed primarily by whether the latent prior is preserved and whether the generated observations remain in distribution with respect to the learned legitimate manifold. Under the ideal flow model, this objective is addressed directly by latent sign modulation through Proposition 1 and Corollary 1. Communication reliability, by contrast, is governed by the local geometry of the learned flow, by the stability of the selected coordinates as binary sign channels after observation-domain perturbation, and by the receiver’s ability to aggregate residual soft evidence across many such channels.

This distinction has an important methodological consequence. Preserving the latent prior is sufficient to maintain the intended distributional legitimacy of the inner embedding rule, but it does not guarantee that the resulting sign channels are reliable after inverse-flow synthesis, perturbation, and forward-flow recovery. Reliability must therefore be addressed separately through coordinate selection and outer decoding.

The proposed architecture reflects precisely this separation of roles. The inner sign-modulation mechanism is responsible for preserving the learned legitimate distribution under the ideal model, whereas the two-state selector and the CRC-Polar outer code are introduced to strengthen message recovery over the imperfect sign channels induced by the learned flow geometry. In this sense, the framework does not treat legitimacy preservation and reliability enhancement as the same objective; rather, it addresses them through different layers of the overall design.

## 5. Experimental Results and Analysis

### 5.1. Experimental Setup

The legitimate dataset is constructed from IEEE 802.11a [22] NonHT-Data waveforms generated with CBW20, MCS 4, and a PSDU length of 376 bytes. This configuration is chosen so that the resulting NonHT-Data field contains 32 OFDM data symbols. Under this setting, the resulting NonHT-Data field yields a 48×32 complex data grid after OFDM demodulation, where 48 denotes the number of data subcarriers per OFDM symbol.

During preprocessing, each complex data grid is converted into a real-valued vector by concatenating its real and imaginary parts, which gives the native dimension $D_{\mathrm{raw}}=2\times 48\times 32=3072$. To satisfy the architectural requirement of the coupling-based flow implementation, the resulting vector is padded to the model dimension D=4096 using Gaussian noise with standard deviation σ=0.05. The dataset is partitioned into training, validation, and test sets of 30,000, 5000, and 5000 samples, respectively. The validation set is used for checkpoint selection and training monitoring, whereas the test set is reserved for the final evaluation reported in this paper.

The legitimate-signal model is implemented as a Zuko neural spline flow, and the main experimental settings are summarized in Table 1. All neural-network experiments were implemented in a Python 3.10 Conda environment using PyTorch v2.5.0+cu124 and Zuko v1.5.0. The models were trained with the AdamW optimizer implemented in PyTorch. The CRC-Polar encoder and CA-SCL decoder were implemented in-house in the Python codebase. MATLAB R2024b was used to generate the IEEE 802.11a resource-grid dataset. The uncoded reference baseline and the CRC-Polar coded scheme share the same keyed reference latent template and the same two-state robust selector. In the uncoded baseline, each message bit is conveyed by an odd-sized repetition group and recovered by hard sign voting. In the coded scheme, repetition voting is replaced by CRC-8, Polar coding, key-driven interleaving, reliability-weighted LLR extraction, and CA-SCL decoding. Unless otherwise stated, the selector first forms a candidate set and then performs perturbation-and-recovery probing on a subset of coordinates to estimate coordinate-wise sign stability.

### 5.2. Model and Pipeline Validation

Before turning to the main communication results, the learned legitimate-signal model and the implementation pipeline are validated under controlled conditions.

**Figure 3:**
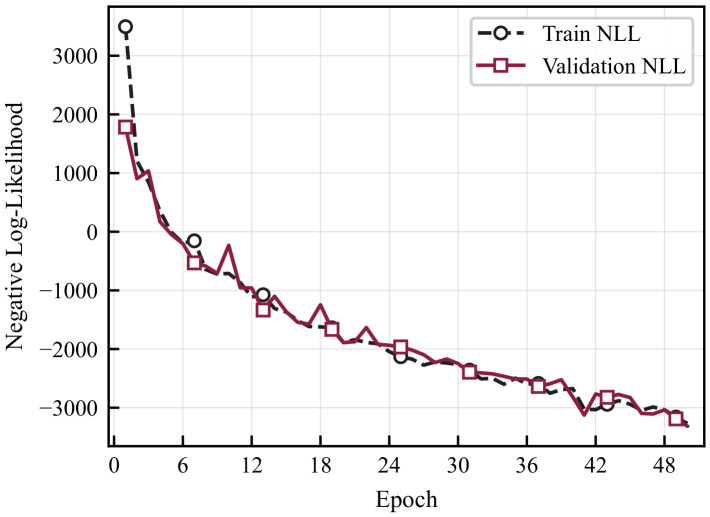
Training and validation negative log-likelihood (NLL) over 50 epochs. Both curves converge stably, and the small gap between them indicates that the learned flow generalizes to unseen legitimate OFDM samples.

The fidelity of the learned legitimate-signal model is first examined both quantitatively and qualitatively. Figure 3 shows the negative log-likelihood (NLL) on the training and validation sets over 50 epochs. Both curves decrease stably and converge without divergence, with the best checkpoint achieving a validation NLL of −3314.68 and a held-out test NLL of −3307.51. The close agreement between these values indicates that the flow generalizes well and captures the high-probability region of the legitimate OFDM observation distribution without evident overfitting.

**Figure 4:**
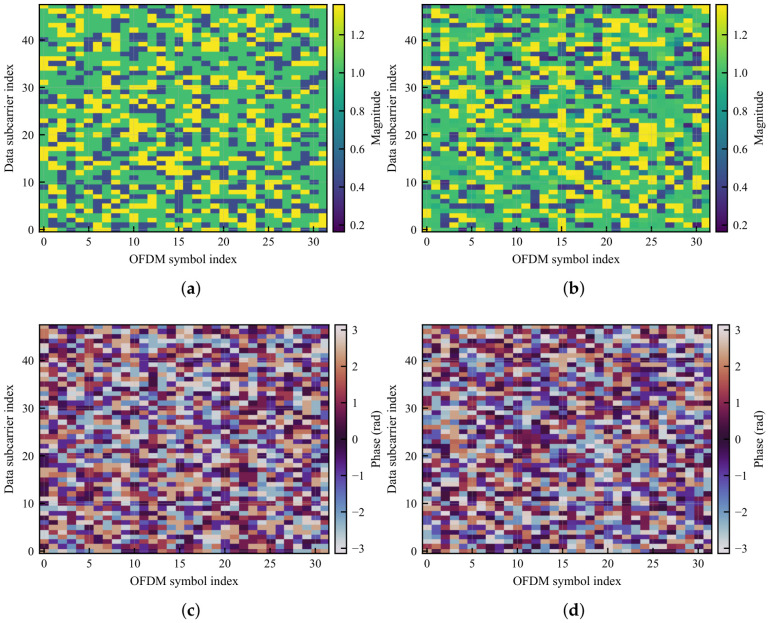
Magnitude and phase diagnostics of the learned OFDM manifold. (**a**) Legitimate magnitude. (**b**) Generated magnitude. (**c**) Legitimate phase. (**d**) Generated phase. The generated sample preserves the main amplitude and phase characteristics of the legitimate data.

Figure 4 further compares the magnitude and phase grids of a legitimate held-out sample with those of a sample synthesized by the trained inverse flow G(·). The generated sample reproduces the discrete amplitude levels associated with the underlying QAM structure as well as the broad phase distribution observed in the legitimate data. These qualitative diagnostics support the view that the learned flow captures the structured geometry of the OFDM data-grid manifold.

The implementation pipeline is then checked before perturbations in the native observation domain are introduced. In the noiseless round-trip setting, the mapping $\tilde{z}\to G\left(\tilde{z}\right)\to F\left(G\left(\tilde{z}\right)\right)$ remains exact up to numerical precision, which confirms the functional consistency of the flow implementation and the correctness of the latent sign-modulation and recovery pipeline. In a further controlled test conducted in the standardized observation space, both coded configurations maintain near-zero BER down to low SNR values. This result shows that the coded receiver operates as expected when perturbation does not strongly interact with the nonlinear geometry of the learned flow. Accordingly, the residual errors observed later in the native observation-domain evaluation are more plausibly associated with nonlinear noise reshaping after flow inversion rather than with an inconsistency in the coding or decoding procedure.

### 5.3. Uncoded Baseline and Geometry-Induced Reliability Loss

To quantify the reliability limitation induced by the learned flow geometry, the uncoded repeated-sign baseline is evaluated under de-normalized sample-space perturbation. This baseline uses the same keyed reference template and the same two-state robust selector as the coded system, but maps the 16 message bits to repetition groups of size r=9 and recovers them by hard sign voting.

Table 2 reports the results over the SNR range from 0 to 20 dB. The table reports 95% confidence intervals under the same $10^{5}$-frame evaluation protocol used for the BER/goodput plots. In particular, the BER increases from 0.0566 with 95% CI [0.0562,0.0570] at 5 dB to 0.0997 with 95% CI [0.0993,0.1002] at 20 dB. The corresponding goodput stays close to 15 bits per frame and does not exhibit a consistent improvement with increasing SNR. This behavior is consistent with the analysis in Section 4: after de-normalization, additive perturbations in the native observation scale interact with the local geometry of the learned flow and produce coordinate-dependent as well as state-dependent latent distortions. As a result, the effective sign reliability does not improve proportionally with the nominal noise reduction. Over the tested SNR range, the uncoded inner sign channel therefore remains geometry-limited in de-normalized sample space and stays in the $10^{-2}$ BER regime despite two-state robust selection.

**Figure 5:**
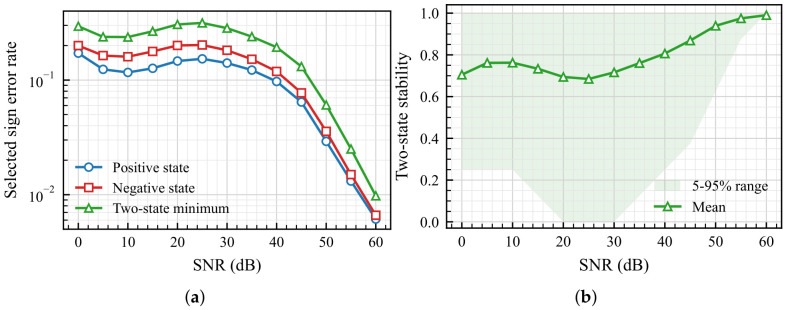
Geometry analysis of the uncoded non-monotonic reliability behavior. (**a**) Selected-coordinate sign error rate for the positive state, negative state, and conservative worst-case two-state stability. (**b**) Two-state selected-coordinate stability with the 5–95% range. The high-SNR points show gradual recovery from the intermediate-SNR reliability trough.

**Figure 6:**
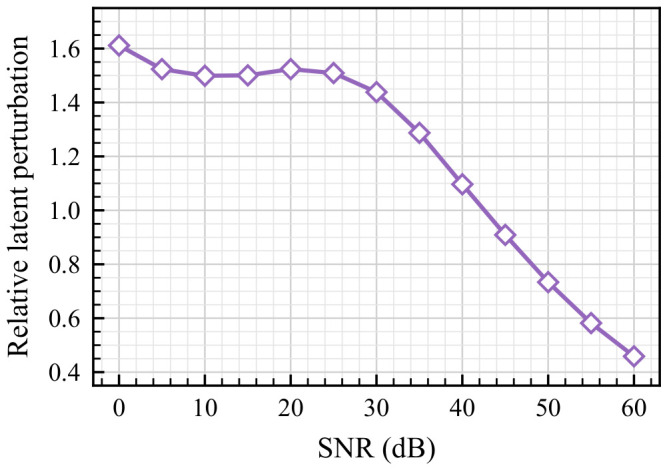
Relative latent perturbation of the selected coordinates. The intermediate-SNR region shows the largest normalized latent displacement after inverse-flow synthesis and forward-flow recovery, which is consistent with the reliability trough in Figure 5.

The non-monotonic uncoded behavior is analyzed through the stability of the selected latent coordinates after inverse-flow synthesis, observation-domain perturbation, and forward-flow recovery. For each SNR point, both positive and negative embedding states are probed, and the selected-coordinate sign error rate and two-state stability are recorded. Figure 5a shows that the selected-coordinate sign error rate is elevated in the intermediate-SNR region and then decreases gradually at high SNR. Figure 5b shows the corresponding two-state stability. The conservative two-state stability decreases from approximately 0.763 at 10 dB to 0.694 at 20 dB, while the lower tail collapses to zero around 20–25 dB. High-SNR points at 35, 45, 50, and 55 dB show that the recovery toward the 60 dB point is gradual rather than an isolated artifact. The relative-latent-perturbation results in Figure 6 show a matching intermediate-SNR increase in normalized latent displacement, providing an additional diagnostic for the geometry-based explanation. These observations suggest that the uncoded anomaly is an intermediate-SNR finite-noise reliability trough associated with state-dependent flow geometry rather than a monotonic high-SNR degradation.

### 5.4. Coded System Performance

The coded scheme addresses the geometry-induced reliability limitation of the uncoded inner sign channel by replacing repetition-based hard voting with CRC-Polar outer coding over the same prior-preserving latent sign modulation. This subsection reports the main coded results under de-normalized sample-space perturbation, including the comparison with the uncoded baseline, the waterfall behavior of the coded link, and the low-SNR verification.

The coded waterfall is evaluated using $10^{5}$ frames per SNR point for both $N_{\mathrm{polar}}=64$ and $N_{\mathrm{polar}}=128$, with exact 95% confidence intervals. For the 16-bit covert payload, each reported operating point contains $1.6\times 10^{6}$ evaluated message bits. Operating points with no observed errors are therefore reported through finite-sample upper confidence bounds rather than as asymptotic zero-error results. Relative to the uncoded baseline in Table 2, the improvement within the tested regime is considerable. At 5 dB, the uncoded BER is $5.66\times 10^{-2}$, whereas the $N_{\mathrm{polar}}=128$ coded system has no observed message-bit errors over $1.6\times 10^{6}$ evaluated message bits. At 20 dB, where the uncoded BER increases to $9.97\times 10^{-2}$, no coded message-bit errors are observed under the same $10^{5}$-frame protocol. The clean-sample flow score of the coded samples remains close to that of the uncoded system, which indicates that the reliability gain is not accompanied by a visible departure from the learned legitimate manifold.

**Figure 7:**
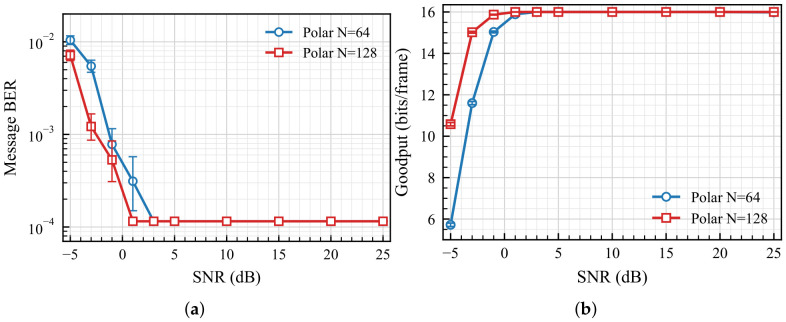
Coded performance under de-normalized sample-space perturbation using $10^{5}$ frames per SNR point for both tested Polar block lengths. (**a**) Message BER versus SNR for $N_{\mathrm{polar}}=64$ and $N_{\mathrm{polar}}=128$; zero-observed BER points are plotted with the corresponding series markers at their finite-sample 95% upper confidence bounds. (**b**) Goodput with finite-sample uncertainty bounds. The longer code reaches the low-BER region and full payload at lower SNR.

Figure 7 shows the message BER and goodput of the coded system versus SNR for $N_{\mathrm{polar}}=64$ and $N_{\mathrm{polar}}=128$. Both coded configurations improve with increasing SNR, while the longer code exhibits a faster transition into the low-BER region. With the added confidence bounds, the conservative conclusion is that the $N_{\mathrm{polar}}=128$ configuration reaches the finite-sample no-observed-error regime earlier than the $N_{\mathrm{polar}}=64$ configuration and maintains full goodput across the moderate-SNR operating range. This reliability advantage also translates into higher goodput: the $N_{\mathrm{polar}}=128$ configuration reaches the full payload of 16 bits per frame at a substantially lower SNR. Although the shorter code selects only 64 coordinates and therefore retains stronger per-coordinate stability, the longer code benefits from additional coding redundancy and achieves the best overall message-level reliability among the tested coded configurations.

Table 3 gives detailed $10^{5}$-frame coded results for the $N_{\mathrm{polar}}=128$ configuration in the low-BER operating region; the $N_{\mathrm{polar}}=64$ coded curve in Figure 7 is obtained with the same $10^{5}$-frame protocol. At 1 dB, the measured BER is $1.5\times 10^{-5}$ with 95% CI $\left[9.61\times 10^{-6},2.23\times 10^{-5}\right]$, which places this operating point in a low-BER but nonzero-error regime. At 3 and 5 dB, no message-bit errors are observed over $1.6\times 10^{6}$ evaluated message bits per SNR point, yielding an exact 95% upper BER bound of $2.31\times 10^{-6}$. The zero-observed-error points are therefore reported as finite-sample upper bounds rather than as asymptotic zero-error claims.

At low SNR, both coded configurations degrade in both BER and goodput. At −5 dB, the $N_{\mathrm{polar}}=128$ configuration yields a message BER of $7.16\times 10^{-3}$, an erasure rate of 0.3315, and a goodput of 10.58 bits per frame, whereas the $N_{\mathrm{polar}}=64$ configuration degrades further to a BER of $1.04\times 10^{-2}$, an erasure rate of 0.632, and a goodput of 5.72 bits per frame. These results confirm that sufficiently adverse conditions still cause decoding failure, with CRC-detected frame erasure remaining the dominant failure mode rather than undetected bit error, consistent with the expected behavior of CA-SCL decoding below its operating threshold.

### 5.5. Residual Frequency-Offset Robustness

To assess physical-layer robustness beyond additive observation noise, the coded system is evaluated under residual carrier-frequency offset, a representative synchronization impairment in OFDM receivers. The impairment is applied as a residual phase rotation across the resource-grid observations with normalized offsets $\epsilon_{\mathrm{cfo}}\in \left\{0,0.001,0.003,0.005\right\}$. Each residual-CFO BER/goodput point uses the same $10^{5}$-frame evaluation protocol as the main coded reliability curves. This experiment remains an observation-domain robustness assessment rather than a complete over-the-air RF-chain validation.

**Figure 8:**
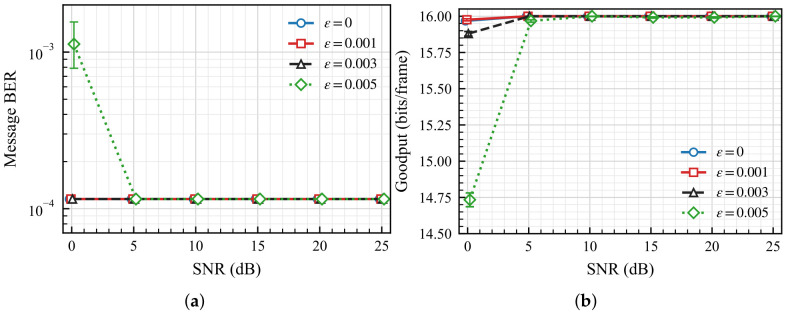
Coded robustness under residual carrier-frequency offset using $10^{5}$ frames per SNR–offset point. (**a**) Message BER with 95% confidence bounds; zero-observed BER points are plotted at the corresponding upper confidence bounds using the corresponding series markers. (**b**) Goodput with finite-sample uncertainty bounds. Overlapping markers indicate that different residual-CFO settings yield identical or nearly identical BER/goodput values at the corresponding SNR points. The strongest tested residual offset mainly affects the 0 dB operating point.

Figure 8 reports the BER and goodput of the $N_{\mathrm{polar}}=128$ coded scheme under the tested residual offsets, with confidence bounds computed from the $10^{5}$-frame trials. The strongest tested offset, $\epsilon_{\mathrm{cfo}}=0.005$, mainly degrades the very-low-SNR operating point: at 0 dB, the BER rises to $1.125\times 10^{-3}$ and the erasure rate to $7.8\times 10^{-2}$. At 5 dB and above, the coded link remains close to full-goodput operation for all tested offsets. These results suggest that the coded mimicry link is most sensitive to residual synchronization error near its low-SNR threshold, while the moderate-SNR operating region remains robust.

### 5.6. Willie-Side Legitimacy Evaluation

Willie is modeled as an observation-domain legitimacy detector. Given a post-demodulation OFDM data-grid observation, Willie decides whether the sample is statistically consistent with the legitimate observation class or whether it is more likely to have been produced by a non-legitimate generation mechanism. The Willie-side evaluation is therefore implemented as a set of separate binary classification tasks in the common observation-domain representation used throughout this paper, each comparing perturbed legitimate observations against one covert or baseline construction.

For each SNR point, three separate Willie-side binary classification experiments are conducted, one for each negative-class construction. In every case, the positive class consists of 5000 perturbed legitimate held-out samples, and the negative class consists of 5000 perturbed samples from the corresponding covert or baseline construction at the same SNR. Both classes are passed through the same de-normalized sample-space perturbation model before classification. The three negative-class constructions are (i) proposed covert, generated by the full proposed scheme; (ii) random-coordinate latent embedding, which retains latent embedding but replaces robustness-aware coordinate selection with random coordinate choice; and (iii) coarse OFDM-format baseline, which preserves only coarse OFDM-format characteristics without learned-manifold matching. For each binary comparison, the data are split into 70% training and 30% test partitions, and the area under the receiver operating characteristic curve (AUC) on the held-out test set is reported.

Table 4 reports the Willie-side AUC values over the SNR range from 0 to 20 dB. The proposed scheme and the random-coordinate latent embedding baseline both remain in a relatively low-discriminability regime, with AUC values between 0.52 and 0.61, indicating that both learned-latent constructions are difficult to separate from legitimate observations under the trained Willie classifier. By contrast, the coarse OFDM-format baseline is much more easily detected, with AUC increasing from 0.72 at 0 dB to 0.91 at 20 dB. This gap shows that coarse OFDM-format resemblance alone is insufficient to achieve legitimacy under the adopted detector.

A direct comparison between the proposed scheme and the random-coordinate latent embedding baseline shows that their Willie-side detection performance is broadly similar across the tested SNR range. The corresponding AUC values remain close at all SNR points, which indicates that introducing robustness-aware coordinate selection does not make the covert observations substantially easier to detect under the trained Willie classifier. Together with the communication results in Section 5.4 and Section 5.7, this comparison shows that the proposed scheme improves communication reliability over random-coordinate latent embedding without introducing an evident Willie-side detectability penalty. The main Willie-side distinction therefore lies not between the two learned-latent constructions, but between both of them and the coarse OFDM-format baseline, which is much more readily distinguishable from legitimate observations.

**Figure 9:**
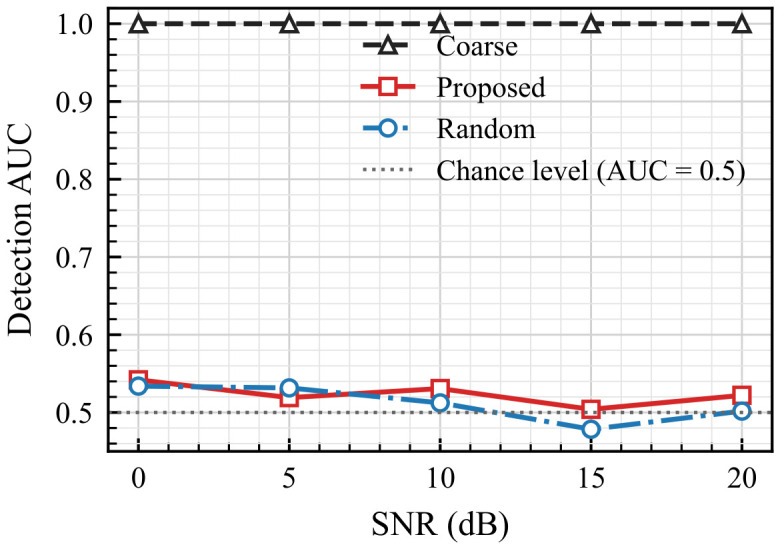
Flow-density typicality Willie detection. The gray dotted horizontal line marks chance-level detection with AUC =0.5. AUC close to 1 indicates easy detection. The proposed covert scheme and the random latent baseline remain close to chance level, while the coarse OFDM-format baseline is highly distinguishable.

Because the trained normalizing flow provides exact density evaluation, Willie is also evaluated with a one-class typicality statistic derived from the learned legitimate density. For an observed sample y, the detector uses

(19) $$T\left(y\right)=\log p_{\theta }\left(y\right),\;{\hat{H}}_{1}\;\mathrm{if}\;T\left(y\right)<\tau_{W},$$

where the threshold $\tau_{W}$ is swept on held-out legitimate samples to generate the ROC curve. This flow-density typicality test identifies samples that fall outside the low-likelihood tail of the learned legitimate distribution and provides a generative counterpart to the discriminative Willie classifier. Figure 9 reports the corresponding AUC values. The proposed covert scheme remains close to chance-level detection, with AUC values between 0.504 and 0.542 over the tested SNR range. The random-coordinate latent baseline behaves similarly. In contrast, the coarse OFDM-format baseline is highly distinguishable, with AUC approximately equal to 1.0 at all tested SNR points. This result indicates that the proposed learned-manifold construction remains difficult to distinguish even when Willie directly uses the trained flow density.

### 5.7. Selector Ablation

The effect of coordinate selection is examined through a matched-condition ablation under a fixed trained flow model, common perturbation settings, identical decoding configurations, and the same finite-sample evaluation protocol. Four selection rules are considered: random coordinate selection, magnitude-only selection, one-state robustness-aware selection, and the proposed two-state robustness-aware selection. The ablation is conducted for both the uncoded system and the CRC-Polar coded system in order to determine whether the final reliability gain arises solely from outer coding or also from the construction of stronger latent sign channels. This comparative selector analysis is separate from the confidence-bounded coded SNR sweeps and is used to isolate the contribution of the coordinate-selection rule.

**Figure 10:**
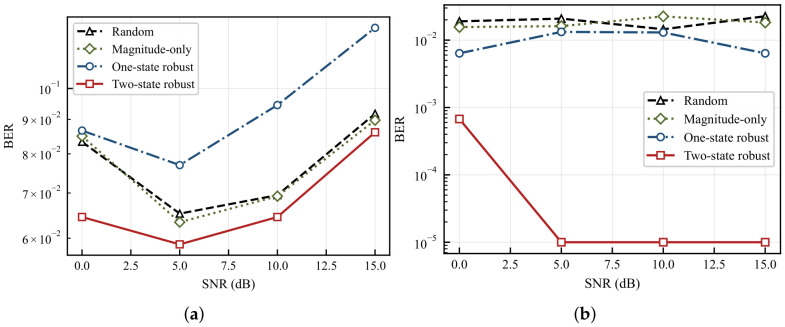
Selector ablation under the common experimental configuration and finite-sample evaluation protocol. (**a**) Uncoded BER versus SNR for random, magnitude-only, one-state robust, and two-state robust selection. (**b**) Coded BER versus SNR under the same selection rules. Zero-observed coded points are interpreted as finite-sample observations rather than asymptotic zero-error claims. The proposed two-state selector yields the lowest uncoded BER and the strongest coded reliability across the tested SNR range.

Figure 10 summarizes the resulting BER trends. Figure 10a reports the uncoded results and therefore reflects the intrinsic quality of the selected latent sign channels. Among the four rules, the proposed two-state selector consistently yields the lowest uncoded BER over the tested SNR range. Its BER is $6.45\times 10^{-2}$ at 0 dB, decreases to $5.88\times 10^{-2}$ at 5 dB, returns to $6.45\times 10^{-2}$ at 10 dB, and increases to $8.61\times 10^{-2}$ at 15 dB. By contrast, random selection and magnitude-only selection remain in the range of approximately $6.3\times 10^{-2}$ to $9.2\times 10^{-2}$, whereas one-state robust selection performs worst overall and reaches $1.23\times 10^{-1}$ at 15 dB. These results indicate that large latent magnitude alone is insufficient for reliable embedding, and that single-state probing is not well aligned with a binary sign channel whose two covert-bit states must both remain reliable after perturbation and recovery.

Figure 10b reports the coded results under the same selection rules. The separation among the four selectors becomes substantially more pronounced once outer coding is introduced. The proposed two-state selector yields a coded BER of $6.75\times 10^{-4}$ at 0 dB and no observed message-bit errors at 5, 10, and 15 dB under the ablation protocol. In contrast, one-state robust selection remains in the range from $6.38\times 10^{-3}$ to $1.33\times 10^{-2}$, while random and magnitude-only selection stay near the $10^{-2}$ regime throughout the tested SNR range. The coded ablation suggests that the final reliability gain cannot be attributed to the CRC-Polar outer code alone. Rather, the proposed two-state selector provides a stronger set of latent sign channels, and the outer code then converts that improved channel set into no observed message-bit errors over the moderate-SNR region under the same ablation protocol.

### 5.8. Complexity, Deployment Scope, and Limitations

The proposed framework consists of components with distinct computational and deployment roles. The normalizing flow is trained offline and is used online for inverse synthesis at Alice and forward recovery at Bob. The two-state coordinate selector is deterministic given the shared key, frame index, trained flow, and public selector parameters, but it requires repeated perturb-and-recover probing; in the tested configuration, it uses a candidate pool of 4096 coordinates, a probe pool of 512 coordinates, and four probe trials. This cost is associated with robust coordinate selection rather than with payload transmission itself. The CRC-Polar layer adds CA-SCL decoding complexity at Bob, with list size 8 in the experiments. On the adversarial side, the flow-density typicality test requires Willie to evaluate the trained flow likelihood and provides a likelihood-based complement to the discriminative classifier. Table 5 summarizes the main computational costs and deployment roles of these components.

The analysis remains within the post-demodulation OFDM resource-grid observation domain. The residual-CFO experiment characterizes a representative synchronization-related impairment within this domain, but it does not model the complete over-the-air RF chain. In particular, explicit fading-channel propagation, timing recovery, carrier recovery, oscillator nonidealities, and hardware nonlinearities are outside the present observation-domain evaluation. Full RF-chain validation is therefore left for future work.

## 6. Conclusions

This paper presents a flow-guided framework for mimicry covert communication over learned legitimate OFDM signal manifolds. The analysis is conducted in the post-demodulation resource-grid observation domain; the framework does not yet model a complete over-the-air RF waveform chain. Within this scope, by modeling legitimate OFDM observations with a normalizing flow and embedding covert information through latent sign modulation, the framework preserves the Gaussian latent prior exactly and therefore preserves the learned legitimate distribution under the ideal model. The analysis and experiments further show that observation-domain perturbations interact with the local flow geometry and create a pronounced reliability bottleneck that is largely hidden in noiseless and normalized-space tests.

To address this bottleneck, the framework combines the prior-preserving inner embedding with a two-stage, two-state robustness-aware coordinate selector and a CRC-Polar outer code with key-driven interleaving and reliability-weighted soft decoding. The resulting coded system yields a considerable operational gain over the strongest uncoded reference baseline within the tested regime while remaining sensitive to sufficiently adverse conditions, as confirmed by the low-SNR evaluation. The reliability measurements and geometry diagnostics associate the uncoded anomaly with an intermediate-SNR two-state stability trough. Under the adopted observation-domain model, the coded construction also retains robustness to moderate residual carrier-frequency offsets and remains close to chance-level detection under the flow-density typicality test. Taken together, these results indicate that legitimacy preservation and communication reliability are related but distinct design objectives in learned-manifold mimicry covert communication. The inner sign rule addresses the former, whereas geometry-aware selection and outer coding address the latter. Future work will focus on full over-the-air RF validation, cross-configuration generalization over MCS and payload settings, fading-channel models, alternative generative embedding baselines, and communication-aware regularization of flow geometry.

## Figures and Tables

**Table 1:** Main experimental configuration.

Item	Configuration
Dataset generation	IEEE 802.11a NonHT-Data waveforms generated with CBW20, MCS 4, and PSDU length 376 bytes; OFDM demodulation yields a 48×32 complex data grid for each sample.
Representation	Real–imaginary concatenation with native dimension $D_{\mathrm{raw}}=3072$, padded to D=4096 using Gaussian noise with standard deviation σ=0.05.
Data split	Training/validation/test =30,000/5000/5000.
Flow model	Zuko neural spline flow with 8 transforms, 2 coupling passes, hidden widths of 512 and 512; AdamW optimizer, learning rate $10^{-3}$, batch size 256, 50 epochs, and additive dequantization noise.
Transmission schemes	Uncoded repeated-sign baseline and CRC-Polar coded scheme using the same keyed reference latent template and two-state robust selector. The coded scheme uses CRC-8, Polar block lengths 64 and 128, key-driven interleaving, and in-house CA-SCL decoding with list size 8.
Selector setup	Candidate pool size 4096, probe pool size 512, minimum latent-amplitude threshold 1.8, probe SNR 10 dB, and 4 probe trials per evaluated coordinate. Coordinates are ranked by the first-stage score in (7) and by the final two-state reliability $r_{i}=\min\left\{p_{i}^{\left(+\right)},p_{i}^{\left(-\right)}\right\}$.
Soft decoding	CA-SCL decoding uses CRC-8, list size 8, LLR scale β=1.0, and clipping threshold $L_{\max}=20.0$.
Evaluation size	All BER/goodput evaluations reported in the reliability and robustness plots use $10^{5}$ frames per reported SNR or SNR–offset operating point. With a 16-bit covert payload, each operating point contains $1.6\times 10^{6}$ evaluated message bits. Finite-sample 95% confidence intervals are reported for the BER estimates, with the corresponding uncertainty shown on the BER and goodput plots.
Noise models	Noiseless round-trip, normalized-space AWGN, de-normalized sample-space AWGN, and residual carrier-frequency offset as a representative OFDM synchronization impairment.
Reported metrics	Message BER, erasure rate, goodput, clean-sample log-likelihood diagnostics, finite-sample confidence intervals, residual-CFO robustness, and Willie-side AUC under classifier-based and flow-density typicality tests.

**Table 2:** Uncoded reference baseline under de-normalized sample-space perturbation with 95% confidence intervals. Each SNR point uses $10^{5}$ frames.

SNR (dB)	BER	95% CI	Goodput (Bits/Frame)
0	0.0620	[0.0616, 0.0623]	15.01
5	0.0566	[0.0562, 0.0570]	15.09
10	0.0653	[0.0649, 0.0656]	14.96
15	0.0832	[0.0828, 0.0837]	14.67
20	0.0997	[0.0993, 0.1002]	14.40

**Table 3:** Detailed $10^{5}$-frame coded performance for the $N_{\mathrm{polar}}=128$ configuration under de-normalized sample-space perturbation. Each SNR point corresponds to $1.6\times 10^{6}$ evaluated message bits for the 16-bit payload.

SNR (dB)	BER	95% CI	Erasure Rate	Goodput (Bits/Frame)
−3	$1.625\times 10^{-3}$	$\left[1.563,1.689\right]\times 10^{-3}$	$7.125\times 10^{-2}$	14.834
−1	$2.00\times 10^{-4}$	$\left[1.787,2.232\right]\times 10^{-4}$	$5.49\times 10^{-3}$	15.90896
1	$1.50\times 10^{-5}$	$\left[9.61,22.32\right]\times 10^{-6}$	$1.40\times 10^{-4}$	15.99752
3	0	$\left[0,2.31\right]\times 10^{-6}$	0	16.000
5	0	$\left[0,2.31\right]\times 10^{-6}$	0	16.000

**Table 4:** Willie-side legitimacy evaluation in the adopted observation domain. Each reported value is the AUC of a separate binary classification test that distinguishes perturbed legitimate observations from one perturbed covert or baseline construction at the indicated SNR under de-normalized sample-space perturbation.

Covert or Baseline Class	0 dB	5 dB	10 dB	15 dB	20 dB
Proposed covert	0.52	0.57	0.61	0.56	0.54
Random-coordinate latent embedding	0.52	0.56	0.58	0.55	0.54
Coarse OFDM-format baseline	0.72	0.80	0.85	0.89	0.91

**Table 5:** Complexity and deployment roles of the main components.

Component	Main Cost	Deployment Role
Normalizing flow	Forward/inverse pass through the trained invertible model	Offline training; online synthesis and recovery
Two-state selector	Candidate probing over 4096 candidates with a 512-coordinate probe pool and 4 probe trials	Shared-key coordinate selection and reliability estimation
CRC-Polar decoder	CA-SCL decoding with list size 8	Receiver-side message recovery and erasure detection
Flow-density Willie typicality test	Flow log-density evaluation	Likelihood-based adversarial legitimacy test

## Data Availability

Data are available from the corresponding author upon request.
